# Effectiveness of *Saccharomyces Boulardii CNCM I-745* probiotic in acute inflammatory viral diarrhoea in adults: results from a single-centre randomized trial

**DOI:** 10.1186/s12876-023-02863-8

**Published:** 2023-07-03

**Authors:** Marcela AG Salazar-Parra, Roberto U Cruz-Neri, Xóchitl AR Trujillo-Trujillo, Juan J Dominguez-Mora, Héctor I Cruz-Neri, Jazmín M Guzmán-Díaz, Mario J Guzmán-Ruvalcaba, Jesús O Vega-Gastelum, Kriscia V Ascencio-Díaz, Maria F Zarate-Casas, Fanny Y González-Ponce, Francisco J Barbosa-Camacho, Clotilde Fuentes-Orozco, Gabino Cervantes-Guevara, Enrique Cervantes-Pérez, Guillermo Alonso Cervantes-Cardona, Ana Olivia Cortés-Flores, Alejandro González-Ojeda

**Affiliations:** 1 Biomedical Research Unit 02, Western National Medical Center, Mexican Institute of Social Security, Avenida Belisario Domínguez #1000, Colonia Independencia, Guadalajara, Jalisco 44340 Mexico; 2 Department of Medical Science, University of Colima, Avenida Universidad #333, Colonia las Víboras, Colima, Colima 28040 México; 3 Surgeon and Coloproctologist, Puerta de Hierro Sur Medical Center, Tlajomulco de Zúñiga, Avenida Adolfo López Mateos Sur #1401, Colonia La Tijera, Jalisco, 45640 Mexico; 4 University of Colima, Universitary Center of Biomedical Research, Colonia Villas de San Sebastián, Avenida 25 de Julio #965, Colima, Colima 28045 Mexico; 5 Puerta de Hierro Medical Center, Tlajomulco de Zúñiga, Avenida Adolfo López Mateos Sur #1401, Colonia La Tijera, Jalisco, 45640 Mexico; 6 High Specialty Geriatric Care Unit, Hospital Civil Fray Antonio Alcalde, Calle Hospital #278, Colonia El Retiro, Guadalajara, Jalisco 44280 México; 7 Departamento de Bienestar y Desarrollo Sustentable, Centro Universitario del Norte, Universidad de Guadalajara, Carretera Federal No. 23, Km. 191, Colotlán, Jalisco C.P. 46200 México; 8 Departamento de Medicina Interna, Centro Universitario de Ciencias de la Salud, Hospital Civil de Guadalajara Fray Antonio Alcalde, Universidad de Guadalajara, Calle Hospital 278, Col. El Retiro, Guadalajara, Jalisco 95100 México; 9 Departamento de Disciplinas Filosófico, Metodológicas e Instrumentales, Centro Universitario de Ciencias de la Salud, Universidad de Guadalajara, Sierra Mojada 950 Edificio “N” planta alta, Col. Independencia, Guadalajara, Jalisco 44340 México; 10 Surgical Oncology, Anker Global Oncology, Av. Juan Palomar y Arias 530, Guadalajara, Monraz, Guadalajara, Jal. Mexico 44670 Mexico

**Keywords:** Probiotics, Viral, Gastroenteritis, Disease recovery, Saccharomyces boulardii, Diarrhoea

## Abstract

**Background:**

Probiotics are effective for treating acute infectious diarrhoea caused by bacteria, but there are inconsistent results for the effectiveness of probiotics for diarrhoea caused by viruses. In this article we want to determine whether *Sb* supplementation has an effect on acute inflammatory viral diarrhoea diagnosed with the multiplex panel PCR test. The aim of this study was to evaluate the efficacy of *Saccharomyces boulardii (Sb)* as a treatment in patients diagnosed with viral acute diarrhoea.

**Methods:**

From February 2021 to December 2021, 46 patients with a confirmed diagnosis of viral acute diarrhoea diagnosed with the polymerase chain reaction multiplex assay were enrolled in a double-blind, randomized placebo-controlled trial. Patients received paracetamol 500 mg as a standard analgesic and 200 mg of Trimebutine as an antispasmodic treatment plus 600 mg of Sb (n = 23, 1 × 109/100 mL Colony forming unit) or a placebo (n = 23) orally once daily for eight days. The improvement in and severity of symptoms were measured using a symptom diary, the Patient Global Impression and the Patient Global Impression of Change scales (days 4 and 8), both answered and recorded by the patient.

**Results:**

Of the 46 patients who completed treatment, 24 (52%) were men and 22 (48%) were women. The average age was 35.6 ± 12.28 years (range 18 to 61 years). The average duration of the evolution of illness at the time of diagnosis was 0.85 ± 0.73 days (maximum 2 days). On day 4 after the diagnosis, 20% reported pain and 2% reported fever, but on day 8, no patient reported pain or fever. On day 4, 70% of patients in the Sb group and 26% in the placebo group reported improvement (P = 0.03), based on the Patients’ Global Impression of Change scale, which assesses patient’s rating of overall improvement. These findings suggest that 3 to 4 days of treatment with *Sb* helped to improve symptoms of diarrhoea caused by a virus.

**Conclusion:**

Treatment with Sb on acute inflammatory diarrhoea of viral aetiology shows no changes regarding the severity of the symptoms; nevertheless, it seems to impact improvement positively.

**Trial registration:**

22CEI00320171130 dated on 16/12/2020, NCT05226052 dated on 07/02/2022.

## Background

Gastrointestinal infections represent a public health problem in developing and industrialized countries [1]. Despite advances and modifications in policies, food safety regulation, and immunization, these diseases affect millions of people each year. Rapid and accurate diagnosis is important to the management and epidemiological surveillance of these infections. The major challenges in the diagnosis of gastrointestinal infections include the wide diversity of associated viral, bacterial, and parasitic pathogens, as well as cultural factors and the identification of the aetiological agents [2].

Acute diarrhoeal disease (ADD) is defined as the expulsion of 3 or more liquid stools, with or without blood, within 24 h, that take the shape of the container in which they are placed. A diarrhoeal episode is one that meets the above criteria and ends when the last day of diarrhoea is followed by at least 48 h of normal stools [3]. Acute diarrhoea lasts less than 14 days, diarrhoea lasting more than 14 days is called persistent diarrhoea, and diarrhoea lasting more than 1 month is called chronic diarrhoea. Severe acute diarrhoea warrants immediate medical evaluation and hospitalization [4].The clinical presentation of viral gastroenteritis ranges from an asymptomatic state to diarrhoea with severe dehydration [2].

Enteric bacterial gastroenteritis can be difficult to differentiate from that of viral aetiology solely based on the clinical presentation mainly because of the presence of leukocytes in stool. In the past, the presence of leukocytes was a specific indication of diarrhoea of bacterial aetiology and was the basis for the diagnosis of acute inflammatory diarrhoea. Hence, laboratory studies are needed to make a specific diagnosis [5].

The methylene blue test is traditionally performed to identify the presence of leukocytes in stool [6]. The multiplex polymerase chain reaction (PCR) test uses an automated system in which the extraction, amplification, and detection of nucleic acid occurs in a single closed pouch. The test panel includes the aetiological identification of bacteria, parasites, and viruses [7].

Probiotics can be bacterial or yeast microbes. Yeast probiotics, such as *Saccharomyces boulardii (Sb)*, are different from bacterial probiotics [3].*Sb* has several different mechanisms of action that can be classified into 3 main areas: luminal action, trophic action, and mucosal anti-inflammatory signalling effects. *Sb* has several benefits such as interference with pathogenic toxins and pathogen adhesion, preservation of cell physiology, interactions with the normal microbiota, and restoration of short-chain fatty acid level. *Sb* can also act as an immune regulator, both within the lumen and systemically [4].

The efficacy of other *Saccharomyces* strains has also been investigated. Unlike other *Saccharomyces* strains, only *Sb* is capable of degrading *Clostridium difficile* toxin, destroying endotoxins of pathogenic *Escherichia coli*, reducing the effects of cholera toxin, and inhibiting the growth of pathogens (such as *Candida albicans*, *Salmonella typhimurium*, *Yersinia enterocolitica*, *Aeromonas haemolysin*) [8].

Normal gut microbiota has many functions but the most pertinent is resistance to colonization, which involves the interaction of many bacterial microflorae and results in a barrier effect against the colonization of pathogens. Factors that disrupt this protective barrier (for example, the use of antibiotics or surgery) increase host susceptibility to pathogen colonization until the normal microbiota can be restored (usually within 6–8 weeks). Probiotics are uniquely qualified to act as a substitute for normal microbiota during this window of susceptibility until recovery. *Sb* does not affect the normal microbiota in healthy human controls, although the normal microbiota is rapidly restored when *S. boulardii* is administered to mice subjected to antibiotic shock or patients with diarrhoea [8].

Based on a systematic review of 27 clinical trials involving 5029 patients, 84% of the treatment groups that received S. boulardii for multiple causes of diarrhea demonstrated significant efficacy and safety. S. boulardii was significantly effective in preventing antibiotic-associated diarrhea, with a relative risk (RR) of 0.50, according to a 2010 meta-analysis of ten randomized, controlled trials in adults [8].

As previously mentioned in this paper, viral gastroenteritis is the leading cause of gastroenteritis, and the administration of probiotics may help to control viral infection. However, the interaction between probiotics and viral gastroenteritis has not been previously evaluated in the Mexican population.

## Materials and methods

### Main objective

The main objective was to determine whether *Sb* supplementation has an effect on acute inflammatory viral diarrhoea diagnosed with the multiplex panel PCR test in private practice.

### Design

This was a randomized, placebo-controlled double-blind study. Initially, patients were randomized in a 1:1 ratio to receive all doses of *Sb*, 3 capsules of *Saccharomyces boulardii* CNCM I-745 200 mg/day, or placebo, 3 starch capsules of 200 mg as placebo/day. Both the placebo and the probiotic capsules had a similar presentation. CMCM I-745 has a concentration of 1 × 109/100 mL Colony forming unit (CFU) [9]. In this study, the rationale for administering 600 mg of S. boulardii to the patients was based on a clinical trial conducted by Hochter et al. [10]. This trial had reported efficacy for acute adult diarrhea in patients who received S. boulardii. Therefore, the dosage selected for this study was consistent with that used in the previously conducted trial, and it was expected to demonstrate similar efficacy.

### Procedure

A total of 46 patients were included and divided into 2 groups, probiotics (n = 23) or placebo (n = 24) groups. The inclusion criteria were age 18 years or more, a positive leukocyte test in stool and a confirmed diagnosis of viral infection by FilmArray™ Multiplex PCR System from bioMérieux, Marcy l’Etoile, France. Patients were included after they provided informed consent.

The exclusion criteria were the presence of known autoimmune disease or inflammatory bowel disease, under immunosuppressive treatment for a known pathology, a confirmed diagnosis of infection by bacteria and/or parasites in the multiplex PCR test whether associated or not associated with viral etiology, previous administration of antibiotic treatment or consumption of any probiotic in the preceding 7 days, known allergy to the probiotic containing *Sb*, clinical positivity to the current operational definition of COVID-19, or no medical insurance. Patients with less than 80% adherence to the indicated probiotic treatment, treatment interruption, or withdrawal of informed consent were eliminated from the analysis.

### Virus identification

The multiplex PCR test was performed using the Gastrointestinal Panel for FilmArray™ Multiplex PCR System. This panel is an automated system in which nucleic acid extraction, amplification, and detection occur in a single closed pouch [11]. The panel includes a total of 22 targets, including bacteria (*Campylobacter jejuni, Campylobacter coli, Campylobacter upsaliensis, Clostridium difficile* (toxin A/B), *Plesiomonas shigelloides, Salmonella, Yersinia enterocolitica, Vibrio parahaemolyticus, Vibrio vulnificus, Vibrio cholerae*); diarrheagenic *E. coli/Shigella (E. coli O157*, enteroaggregative *E. coli*, enteropathogenic *E. coli*, enterotoxigenic *E. coli lt/st, Shiga-like toxin-producing E. coli stx1/stx-2, E. coli O157, Shigella/*enteroinvasive *E. coli*; parasites (*Cryptosporidium, Cyclospora cayetanensis, Entamoeba histolytica, Giardia lamblia*); and viruses (*Adenovirus F 40/41, Astrovirus, Norovirus GI/GII, Rotavirus A, Sapovirus I, II, IV, and V*) [12, 13].

### Questionnaires

The Patient Global Impression scale (PGIs) is the patient-reported outcomes counterpart to the Clinical Global Impressions scale. The PGIs is a 1-item questionnaire that asks the patient to rate the severity of a specific condition. The Patient Global Impression of Change (PGIC) measures the patient’s change in clinical status [15]. Each participant in the study was provided with a patient diary in which they recorded the number of stools using the Bristol stool scale, the presence of pain or fever, and treatment adherence on a daily basis. Patients also self-reported their symptoms using the PGIC and PGI scales in the diary. The patient diaries and scales were evaluated on days 4 and 8 of treatment. The methods used to measure pain in the participants was the visual analogue scale (VAS) for pain. Patients were instructed on how to respond to the VAS. The guidelines for completing the form were followed. A value between 0 and 4 indicates the absence of pain. For this study, pain was considered present at a score greater than 4 [14].

The improvement was measured in two ways: first, on days 4 and 8, using the PGIC and PGIS scales. The patient was deemed to have improved when he or she reported feeling significantly and slightly better. No improvement was considered: unchanged, slightly worse, and significantly worse. With the analysis of the patient’s diary, an improvement was deemed to have occurred at a value of 0 Bristol evacuations grade 6 and 7, as well as the absence of pain or fever reported by the patient.

### Statistical analysis

Means and standard deviations were calculated for the quantitative variables, and frequencies and proportions were calculated for the qualitative variables. Qualitative variables were compared using the chi square test. The Shapiro–Wilk test was used to determine the distribution of the data. The Cochran–Mantel–Hansel statistical test was used to identify the intervening variables. Relative risk and 95% confidence intervals (CIs) were calculated for symptom persistence after the beginning of the treatment and improvement of diarrhoea during treatment. *P* < 0.05 was considered to be significant. The data were transferred to an Excel file, and the statistical analysis was performed using the IBM SPSS Statistics (version 20.0, IBM, Corp., Armonk, NY, USA).

## Results

**Fig. 1:**
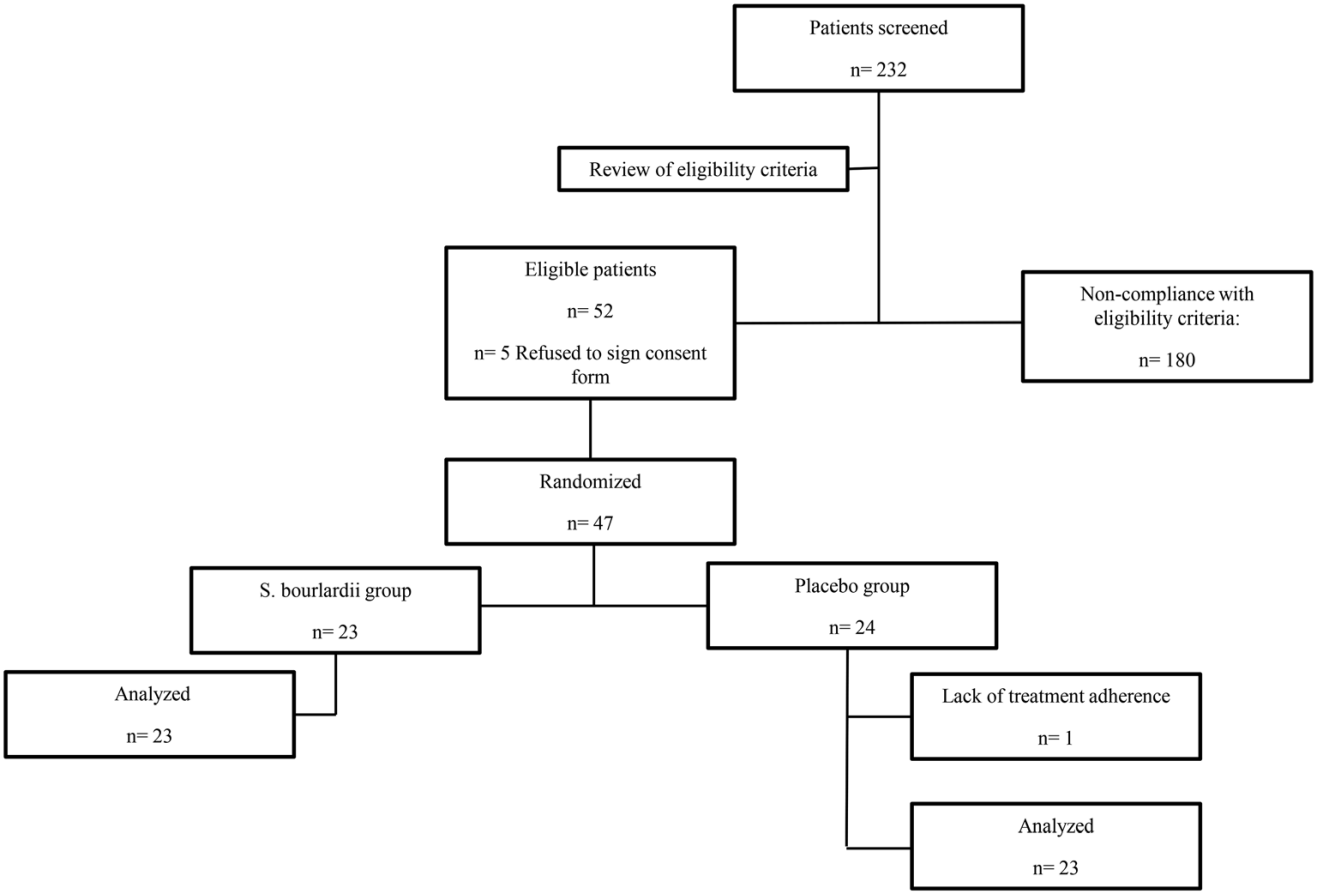
Subjects disposition flow chart

Patient recruitment began on 15 February 2021 and concluded on 27 December 2021. A total of 232 multiplex PCR tests were performed on samples from patients who met the criteria for methylene blue positivity; 52 met the inclusion criteria. Of these patients identified, 47 agreed to continue in the study, but 46 completed the treatment regimen after 1 patient was withdrawn because of lack of treatment adherence (Fig. 1).

The patient characteristics are shown in Table 1. Of the 46 patients who completed treatment, 24 (52%) were men and 22 (48%) were women. The average age was 35.6 ± 12.28 years (range 18 to 61 years). The average duration of the evolution of illness at the time of diagnosis was 0.85 ± 0.73 days (maximum 2 days).

**Table 1:** Baseline characteristics in the *Sb* and placebo groups

	*Saccharomyces boulardii* (n = 23)	Placebo (n = 23)	*P* value
Age, years ± SD	34.43 ± 2.3	36.95 ± 2.7	0.517
Sex, female/male	9/14	13/10	0.238
Days of evolution	1 ± 0.74	0.70 ± 0.70	0.16
Daily bowel movements	7.52 ± 2.3	8.6 ± 2.4	0.12
Abdominal pain	15 (65%)	15 (65%)	1.00
Fever	4 (17%)	4 (17%)	1.00
Cause of diarrhoea			
*Norovirus GI, GII*	15 (65%)	17 (74%)	1.00
*Rotavirus A*	6 (26%)	6 (26%)	
*Sapovirus*	2 (9%)	0 (0.0%)	

*Sb*:*Saccharomyces boulardii*; SD: Standard deviation

The average number of daily bowel movements at the time of diagnosis was 8.02 ± 2.39 (range 3 to 15); 65% of the patients reported feeling pain at the beginning of treatment and 17% of the patients presented with fever. The baseline characteristics did not differ significantly between the *Sb* and placebo groups.

The type of viral pathogens found in patients diagnosed with acute inflammatory diarrhoea of viral aetiology seen in the gastroenterology and coloproctology clinics did not differ between the 2 groups: 65% of the patients in the *Sb* group and 74% in the placebo group presented with *Norovirus* GI/GII, 26.1% of patients in both groups had a positive result for *Rotavirus A*, and 9% in the *Sb* group and no patients in the control group presented with *Sapovirus*. One patient had a combination of *Rotavirus A* and *Adenovirus F40*, and this patient was included in the group with *Rotavirus A*. No patients were shown to be positive for *Adenovirus F40/41* or *Astrovirus*.

As shown in Table 2, on day 4, 16 (70%) of patients in the *Sb* group and 6 (26%) patients in the placebo group reported improvement (*P* < 0.03). For the associated symptoms on day 4, 9 patients (20%) reported pain, and 1 patient (2%) reported fever. On day 8, no patient reported pain or fever. On days 4 and 8, the severity of symptoms, as assessed using the PGIs and PGIC, did not differ between groups. According to the patient dairy, patients reported improvement after 4 ± 1 days in the *S. boulardii* group and after 5 ± 0.95 days in the placebo group (*P* = 0.001).

**Table 2:** Characteristics of the study patients in the *Sb* and placebo groups

	*Saccharomyces boulardii* (n = 23)	Placebo (n = 23)	*P* value	RR (95% CI)
Treatment compliance			0.545	
80% 88% 100%	2 2 19	2 0 21		
Abdominal pain on day 4	4	5	0.500	1.2 (0.38–4.07)
Abdominal pain on day 8	0	0	1.00	
Fever on day 4	1	0	0.500	1.045 (0.95–1.14)
Fever on day 8	0	0	1.00	
Improvement according PGIC scale				
< 4 days	16 (70%)	6 (26%)	0.03	0.16 (0.04–0.55)
> 4 days	7 (30%)	17 (74%)		
Day of improvement mean ± SD	4 ± 1	5 ± 0.95	0.001	
Improvement according to patient dairy				
Day 2	1	0		
Day 3	7	1		
Day 4	8	5		
Day 5	5	12		
Day 6	2	3		
Day 7	0	2		

*Sb: Saccharomyces boulardii;* RR: Relative Risk; CI: Confidence Interval; SD: Standard deviation

## Discussion

Similar to findings reported in the international literature [15, 16], the most frequent viral aetiology found in this adult population with acute viral diarrhoea was *Norovirus GI/GII* followed by *Rotavirus.* Despite evidence in Mexico that the paediatric population younger than 2 years usually presents with viral illness caused by *Rotavirus* [2], and the immune response generated favours less severe disease caused by this virus, our country continues to experience a high incidence of *Rotavirus* infection that requires hospital medical attention. Gonzalez et al. [17], identified *Rotavirus* in 14 of 100 faecal samples collected in children with gastroenteritis in the state of Sonora, Mexico; in our study, 20% of samples exhibited different viruses and 5% were specific for *Rotavirus*.

The mean duration of infection in our study was 4.5 days. The literature reports that the duration of *Norovirus* infection is shorter than the general average, which is 2 to 5 days [2, 18]. Although *Rotavirus* was the second most frequent aetiological agent in our study, the average duration of symptoms was 2 to 7 days, which is similar to that in the general population.

For the symptom severity in our study, no significant difference was obtained after 4 and 8 days of treatment. This finding differs from that reported in a meta-analysis in which the diarrhoea severity score on day 3 of treatment was significantly lower in Sb-treated patients (5.5 ± 6.8) than in the placebo group (6.7 ± 8.7) (P = 0.04) [11].

Although self-reported improvement on days 4 and 8 did not differ between the *Sb* and placebo groups, most patients in the *Sb* group (65%) reported improvement on days 3 and 4, whereas the highest percentage of patients in the placebo group (52%) reported improvement on day 5. This finding is similar to that reported for pooled data from 17 trials that found that *Sb* reduced the mean duration of diarrhoea by 19.7 h [19].

This decrease in the duration of symptomatology may be relate to the mechanisms of action of probiotics, such as an increase in the production of short-chain fatty acids in colonocytes, reduction in the permeability of the intestinal barrier, or a decrease in the invasion of microorganisms [20]. For example, *Sb* induces high levels of IgA and interleukin 10 in the bowel, and these participate in the immunomodulatory response to infection. Specific beneficial effects have been reported in the treatment of children with ADD, prevention of *C. difficile* infections, and prevention of diarrhoea associated with the use of antibiotics, including anti-toxin and anti-inflammatory effects, trophic effects on enterocytes, stimulation of the immune response, increase in disaccharidase levels, elimination of toxins and pathogens, and interference with the bacterial signalling pathways [3].

Most patients in the present study (65%) presented with pain as the primary symptom. It is relevant that only 17% presented fever, which is the most common symptom reported in the literature [16, 18]. Pain is usually the second most common particularly for patients with *Norovirus* as the causal agent.

The primary limitations of this study are the small sample size and the underrepresentation of the pediatric population. The used questionnaires do not collect data on some standard endpoints typically evaluated in diarrhea studies, such as hydration status and associated symptoms such as nausea and vomiting. As a result, it may not provide a complete picture of treatment response and may hinder the ability to interpret study results.

The simple stool examination continues to be the primary test at the time of diagnosis. However, we included only those patients who obtained a positive methylene blue test result, which in clinical practice would suggest an infection caused by toxin-producing bacteria. It is therefore essential to identify specifically the aetiology of the disease.

## Conclusion

In this study, the most frequent aetiology was *Norovirus* GI/GII infection followed by *Rotavirus, Sapovirus*, and the combination of *Rotavirus A* and *Adenovirus F40*. Both the *Sb* and placebo groups exhibited similar symptoms on day 4 after the diagnosis (20% with pain and 2% with fever). On day 8, no patient reported any associated symptom. Treatment with Sb on acute inflammatory diarrhoea of viral aetiology shows no changes regarding the severity of the symptoms; nevertheless, it seems to impact improvement positively. Most patients (70%) in the *Sb* group reported improvement on days 3 and 4; by contrast, in the placebo group, the highest percentage of patients (74%) reported improvement on day 5 (*P =* 0.03). The severity of the symptoms did not differ significantly between the 2 groups on days 4 and 8.

## Data Availability

The datasets used and analysed during the current study are available from the corresponding author on reasonable request.

## Abbreviations

Sb: Saccharomyces boulardii
ADD: Acute diarrhoeal disease
PCR: Polymerase chain reaction
